# Mechanisms behind context-dependent role of glucocorticoids in breast cancer progression

**DOI:** 10.1007/s10555-022-10047-1

**Published:** 2022-06-27

**Authors:** Henriett Butz, Attila Patócs

**Affiliations:** 1 Department of Molecular Genetics and the National Tumor Biology Laboratory, National Institute of Oncology, Budapest, Hungary; 2 Hereditary Tumours Research Group, Hungarian Academy of Sciences-Semmelweis University, Budapest, Hungary; 3 Department of Laboratory Medicine, Semmelweis University, Budapest, Hungary

**Keywords:** Glucocorticoid receptor, NR3C1, Glucocorticoid, Breast cancer, Steroid

## Abstract

Glucocorticoids (GCs), mostly dexamethasone (dex), are routinely administered as adjuvant therapy to manage side effects in breast cancer. However, recently, it has been revealed that dex triggers different effects and correlates with opposite outcomes depending on the breast cancer molecular subtype. This has raised new concerns regarding the generalized use of GC and suggested that the context-dependent effects of GCs can be taken into potential consideration during treatment design. Based on this, attention has recently been drawn to the role of the glucocorticoid receptor (GR) in development and progression of breast cancer. Therefore, in this comprehensive review, we aimed to summarize the different mechanisms behind different context-dependent GC actions in breast cancer by applying a multilevel examination, starting from the association of variants of the GR-encoding gene to expression at the mRNA and protein level of the receptor, and its interactions with other factors influencing GC action in breast cancer. The role of GCs in chemosensitivity and chemoresistance observed during breast cancer therapy is discussed. In addition, experiences using GC targeting therapeutic options (already used and investigated in preclinical and clinical trials), such as classic GC dexamethasone, selective glucocorticoid receptor agonists and modulators, the GC antagonist mifepristone, and GR coregulators, are also summarized. Evidence presented can aid a better understanding of the biology of context-dependent GC action that can lead to further advances in the personalized therapy of breast cancer by the evaluation of GR along with the conventional estrogen receptor (ER) and progesterone receptor (PR) in the routine diagnostic procedure.

**Supplementary Information:**

The online version contains supplementary material available at 10.1007/s10555-022-10047-1.

## The pleiotropic role of GCs and breast cancer

Breast cancer is the most common cancer in women worldwide with an estimated 2.3 million new cases yearly [1]. Histologic classification is evaluated based on the growth pattern (in situ vs. invasive). The most common type (70–80%) is infiltrating duct carcinomas with no special type (IDC), followed by invasive lobular carcinomas (ILC, ~ 10%) [2, 3]. The remaining ones (mucinous, cribriform, micropapillary, papillary, tubular, medullary, metaplastic, and apocrine carcinomas) can be considered rare. Tumor grade (by assessment of histologic differentiation) and stage (TNM, Tumor size, Nodal status, and distant Metastasis) have also important prognostic roles and they are considered in a number of clinical decisions [2]. Early breast cancer accounts for > 90% of all diagnosed breast cancers and despite the availability of different treatment options, ~ 30% of these patients will develop cancer recurrence/progression [4]. Locally advanced or metastatic breast cancers have a median overall survival of approximately ∼3 years and the 5-year survival is only ∼25% [5].

Immunophenotype determined by immunostaining of estrogen receptor (ER), progesterone receptor (PR), and human epidermal growth factor receptor 2 (HER2) provides the basis of targeted therapy selection. Hormone receptors (ER and PR) are expressed in the great majority (∼75%) of all breast cancers and indicates the responsiveness to hormonal therapy [2]. HER2 overexpression is detectable in approximately 15% of breast cancers, due to gene amplification, and it is associated with a more aggressive clinical course and poor prognosis. It is also considered as an important predictive marker indicating the response to anti-HER2 therapy.

Based on global gene expression profile, breast cancer was classified into four subtypes: luminal A, luminal B, HER2-overexpressing, and basal-like breast cancers [2]. Luminal A subgroup is characterized by an immunophenotype of ER + , PR + , and HER2 − , and the proliferation index Ki-67 is low (< 20%). These are typically low-grade tumors with the best prognosis among all subtypes. Luminal B breast cancers are also ER + ones, PR and HER2 status can be both + / − , but Ki-67 index is higher (> 20%). These tend to be higher grade and have a worse prognosis compared to luminal type A tumors [2]. While patients with both luminal type tumors are likely to benefit from hormonal therapy alone, luminal B tumors may be candidates for additional chemotherapy. HER2 overexpressing (HER2 +) subtypes of breast cancer are ER − and PR − , and they likely to be high grade exhibiting an aggressive clinical course. Nevertheless, due to their HER2 positivity, they respond to anti-HER2-targeted therapy which results in an improved outcome. The basal-like tumors (also referred as triple-negative breast cancer showing negative staining of all ER − , PR − , and HER2 −) are characterized by high grade and high proliferation index.

These subgroups usually show good correlation with the immunophenotypic classification, which highlights the significance of the pathological assessment. Indeed, in routine clinical practice, the application of gene expression profile-based classification is limited due to cost and technical challenges [2].

GCs, especially dexamethasone (dex), are routinely administered as adjuvant therapy to manage the side effects of cytotoxic chemotherapy due to their antiemetic effects, energy and orexigenic properties, and to prevent hypersensitivity reactions. However, recently, it has been revealed that dex triggers different effects depending on the breast cancer molecular subtype, which has raised new concerns regarding the generalized use of GC, and suggests that the context-dependent effects of GCs and GC resistance can be taken into potential consideration during treatment design [6-8].

Glucocorticoids (GCs) are steroid hormones synthesized in the adrenal cortex and controlled by the hypothalamic–pituitary–adrenal (HPA) axis. They are classical stress hormones induced by several factors, including psychological stress, infection, inflammation, trauma, toxins, and others. They regulate several cellular processes including metabolism (glucose, protein, lipid, and carbohydrate), immune and inflammatory responses, as well as vascular tone. GCs also widely influence the operation of the central nervous system (arousal, cognition, mood, and sleep), and they have a role in regulation of the circadian rhythm, cell cycle, and programmed cell death [6].

**Fig. 1:**
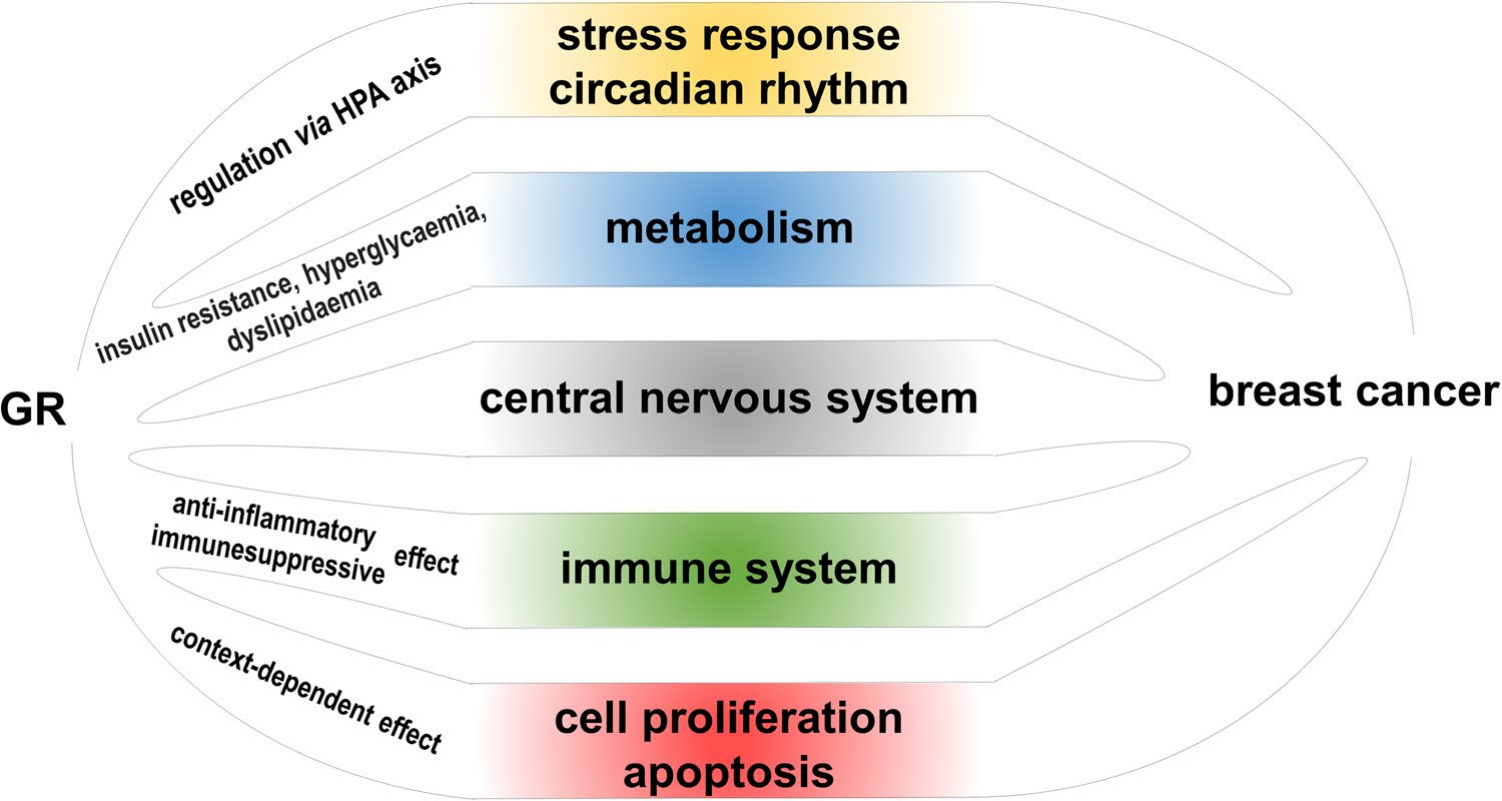
Links between the pleiotropic role of glucocorticoids and breast cancer development and progression. See details in the text

These pleiotropic effects of GCs are involved in the regulation of development of breast tissue, and also in fine tuning of physiological and pathophysiological processes of breast tissue, including breast cancer development (Fig. 1). The dual role of GCs in breast carcinogenesis has been shown in tumor development, cell proliferation, and apoptosis [9]. Chronic stress, disturbed circadian rhythm, inflammation, metabolic syndrome, obesity, depressive behavior, and major depression which have been associated with glucocorticoid effects all have been also linked to increased breast cancer risk and progression [ 7–15].

All these effects should be placed in certain context because the glucocorticoid effects are highly dynamic both in time and space. Related to tumor and metastasis development, the following points especially emphasize the role of GC action in breast cancer: (1) GCs seem to act as both tumor suppressor and tumor promoter in breast tissue in a cell-type specific manner and in a context-dependent way; (2) in the majority of breast cancer cases, tumor development and growth are initially steroid hormone dependent and involve GR crosstalk with other hormone receptors; (3) GCs are routinely administered as adjuvant treatment for the side effects of conventional chemotherapy.

**Fig. 2:**
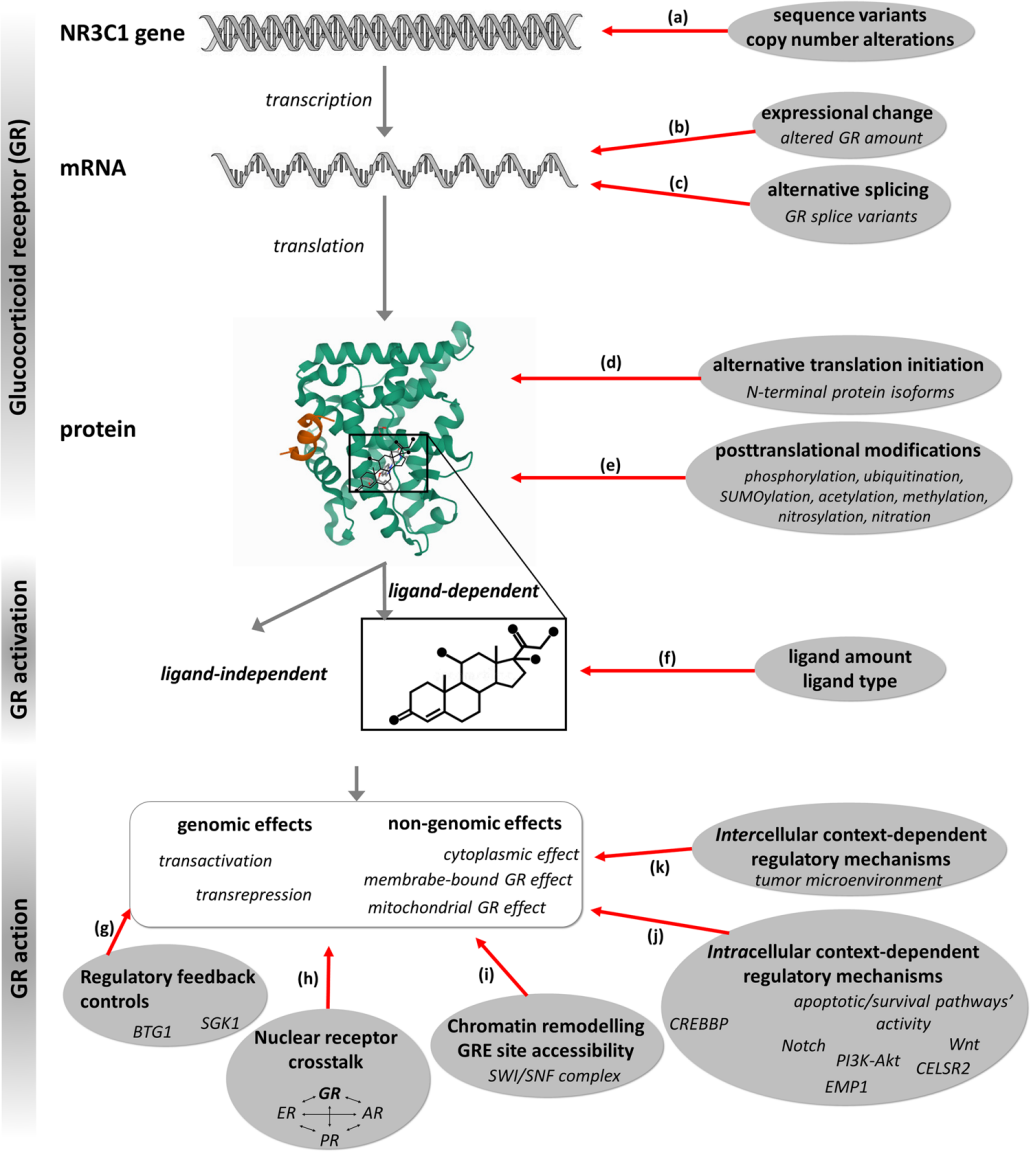
Mechanisms of altered GC response and context dependency assessed in a multilevel approach

In this review, following a brief description of the molecular background of the diverse biological effects of the GR, we summarize the already known and potential factors and mechanisms (Fig. 2) that regulate glucocorticoid action involved in breast carcinogenesis, and discuss the potential therapeutic implications.

## The background of glucocorticoid receptor–mediated biological effects

The GR is expressed almost ubiquitously among human tissues due to the presence of multiple transcription factor-binding sites in its promoter [10]. The detailed structure and action of the glucocorticoid receptor (GR) have been described in excellent reviews [10–12] and is beyond of the scope of this work; however, a brief summary is essential to the understanding of the diverse functions of GR.

### Structure of GR

The GR protein itself consists of three functional domains: the N-terminal (NTD), the central DNA-binding (NBD), and the C-terminal ligand binding (LBD) domains. In addition, a linker or hinge (H) is located between the DNA and ligand binding parts of the receptor. The amino terminal (NTD) domain contains the coding sequence for the strong transactivation domain 1 (AF1, activation function 1) responsible for interaction with co-regulators, chromatin modifiers, and transcription machinery [9, 10, 13]. The DNA binding domain is responsible for the binding to the GR responsive elements (GRE) of the DNA through two zinc finger motifs. In addition, it contains a dimerization and a nuclear localization domain (NLS1) [9, 13-15]. The linker region has role in nuclear translocation and transactivation in addition to its structural function [16]. At the C-terminal part of the receptor, the ligand binding domain can be found, which is responsible for recognizing steroid molecules; a weaker activation function domain (AF-2) and a second nuclear localization domain (NLS2) are also located here [13].

In the absence of ligands, GR is predominantly found in the cytoplasm complexed with accessory proteins (heat-shock proteins and immunophilins), while upon ligand binding, conformational changes occur that lead to receptor activation [17].

### GR action—genomic effects

**Fig. 3:**
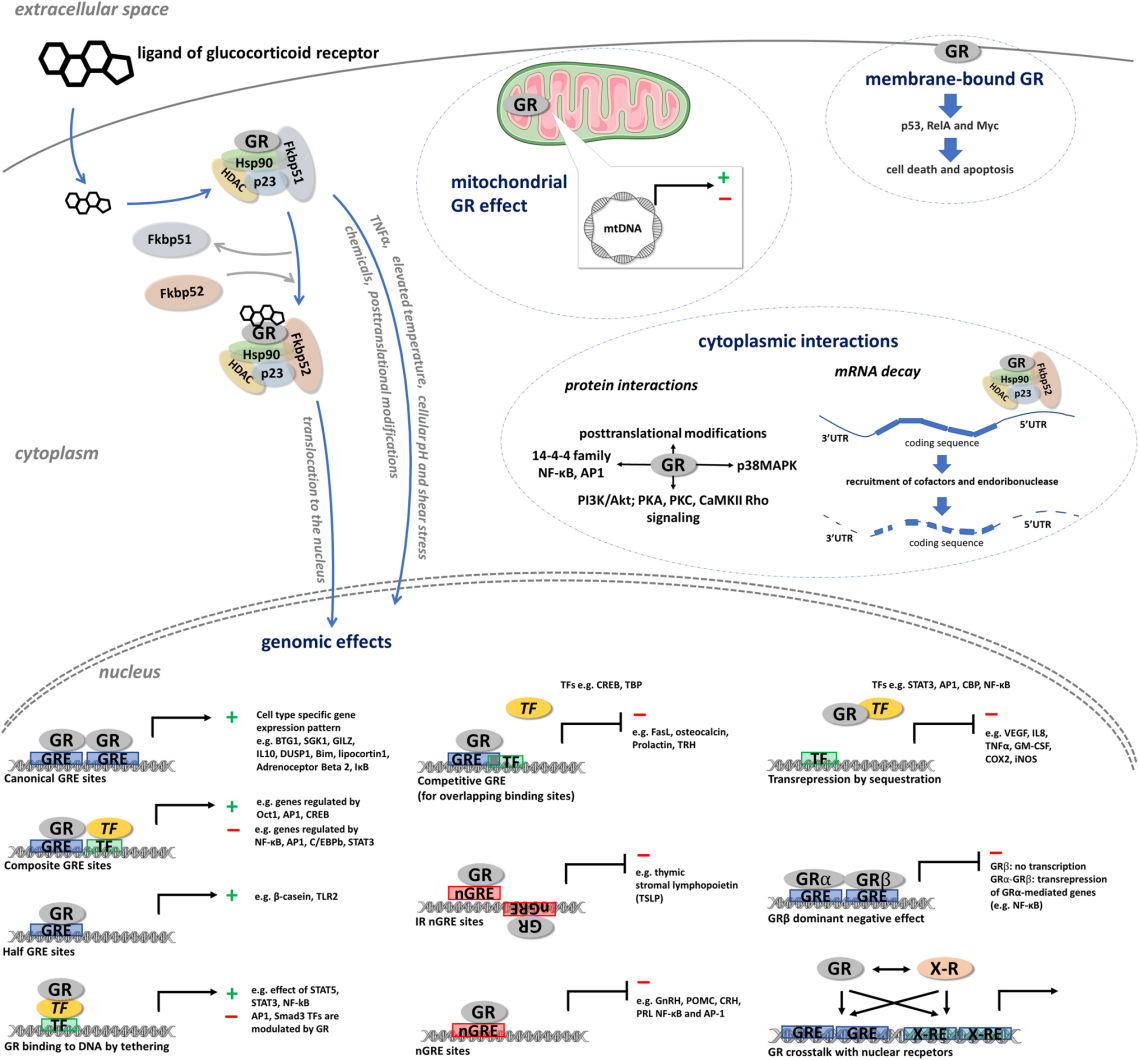
GR activation and GR-mediated mechanisms of action. See details in the text

Following maturation and protein folding, the receptor recruits final chaperones and immunophilins (hsp90 (heat shock protein 90), hsp70 (heat shock protein 70), hsp56 (heat shock protein 56), p23 (protein 23), and FKBP51 (FK506-binding protein 51) and FKBP2 (FKBP prolyl isomerase 2), respectively) increasing its ligand affinity [18, 19]. Upon ligand binding, GR is phosphorylated, leading to conformational changes and surface exposure of the nuclear localization signal (NLS) (Fig. 3) [10, 20]. The ligand bound GR then translocates to the nucleus. The dynamic accessibility of GREs depends on cell type and cellular context, [21]. Upon binding to GRE, GR recruits various nuclear coregulators, both activators or repressors, chromatin remodeling complexes, and histone acetyl transferases regulating chromatin accessibility and GR transcriptional activity [6, 9]. Eventually, GR activation results in enhancement and/or repression of gene transcription, depending on the GRE sequence and promoter.

Several genomic modes of action of the GR have been described and summarized (Fig. 3) [9, 18]. It can bind to GRE directly or through other transcription factors (tethering) and the GR effect highly depends on the GRE type. The main mechanism of GR action is through GR homodimer direct binding to the ***consensus GREs*** [22, 23]. The consensus GREs, containing two palindromic sequences (AGAACAnnnTGTTCT), enable two GR monomers to bind to the DNA molecules. Besides consensus GRE, ***composite or half GRE sites*** have been also described, where the ligand–GR complex can bind as a monomer [22, 24-26]. These composite sites are often located close to other transcription factor sites, modulating each other’s effect. ***Negative GREs*** (nGREs) consist of inverted repeats where the two GR monomers occupy the opposite sites of the DNA, leading to transcriptional repression by recruiting co-repressors and histone deacetylase activity [9, 18, 22, 24, 27, 28]. As GREs can overlap with other transcription factor binding sites in the genome (***competitive sites***), this can lead to competition between ligand–GR complexes and other activated transcription factors. Hence, GR can interfere and decrease the recruitment of other coregulators, leading to a decreased gene expression effect of other transcription factors [9, 18, 22-24].

In addition, GR can bind to DNA indirectly, tethering other transcription factors (e.g., NF-κB (nuclear factor kappa B), AP-1 (activator protein 1), STATs (Signal Transducer And Activator Of Transcription), Oct (octamer transcription factor), NF-1 (neurofibromin 1), C/EBP (CCAAT/enhancer binding proteins), COUP-TFII (COUP transcription factor 2), PPARs (peroxisome proliferator-activated receptors), and LXR (liver X nuclear receptor)) through protein–protein interactions (***tethering sites***), by which it influences their transcriptional activity (Fig. 3) [9, 18, 24, 29].

GR-mediated transactivation activities are realized by the direct binding of GR to GRE sequences, either alone or in association with other transcription factors (TFs). Transrepression is mediated by directly through binding to negative GREs (nGREs) or indirectly through binding to competitive sites or tethering mechanisms [9, 18, 20].

#### Ligand-independent activation

Interestingly, several lines of evidence have demonstrated that GR can also be activated in the absence of ligands [9, 18, 30-32]. Physical factors, such as certain chemicals, elevated temperature, cellular pH, and shear stress, can induce GR activation, hence its nuclear translocation [13, 18]. Increased amounts of receptor posttranslational modifications and the presence of TNFα (tumor necrosis factor alpha) were also shown to induce ligand-independent GR activation [13, 33]. Also, AF-1 located at the NTD of the GR is able to activate target genes in a hormone-independent fashion [34] (Fig. 3).

Altogether, in GC target tissues, GCs influence the expression of a significant amount (roughly 10–20%) of genes by either positive or negative regulation, depending on the cell/tissue type [35, 36]. However, GC-induced gene expression is cell type specific and only a small proportion of genes are commonly activated between different tissues regulated by the tissue-specific chromatin landscape [35].

These genomic effects (i.e., transcriptional changes) of the GR generally occur within a few hours. However, in certain cases, the increase in mRNA expression can be detected 15 min after GC treatment [37].

### GR action—non-genomic effects

The action of transcriptional machinery requires time; therefore, behind the glucocorticoid effects occurring within a few seconds or minutes, other mechanisms have been suggested, compared to the classical genomic effects of the GR [38]. In in vitro experiments, upon GR activation, fast changes of different signaling pathways (e.g., PI3K/Akt (phosphoinositide 3-kinase/AKT serine/threonine kinase 1); PKA (protein kinase A), PKC (protein kinase C), CaMKII Rho (calcium/calmodulin-dependent protein kinase II/Rho kinase)) have been detected [38, 39] (Fig. 3).

It has been suggested that dissociating ***members of the cytoplasmic GR complex*** (chaperones and immunophilins (HSP90, HSP70,HSP56, p23, and FKBP51 and FKBP2)) during receptor activation can also have a role in the GR-mediated fast response regarding MAPK (mitogen-activated protein kinase) signaling modulation [40, 41]. The protein–protein interaction of ligand bound GR with the regulatory subunit of phosphoinositol-3-kinase in endothelial cells activates the protein kinase Akt and mediates further signaling events [39, 40].

Besides the cytoplasm, GR was also shown to be associated to the cell membrane. Membrane-bound GR receptors (mGR) are more related to intracellular signaling pathways mediated by G-protein-coupled receptors [42, 43]. The ***membrane-bound GR*** was proposed to be generated through diverse mechanisms including alternative splicing, as membrane-bound GR and cytosolic GR had a high sequence homology and most probably originate from the same gene [44].

Alongside regulating classical signaling pathways, GR has been identified in ***mitochondria*** and can mediate GR non-genomic effects too. Through GRE-like DNA regulatory elements in the mitochondrial DNA, GR seems to control mitochondrial gene expression, hence cellular energy and metabolism, even in a ligand-independent way [18, 39].

Lately, it has been also revealed that GR is able to regulate mRNA stability by binding the 5′ untranslated region (UTR) of mRNA molecules, a process called GR-mediated mRNA decay, as summarized by Mayayo-Perralta and co-workers [9, 45, 46]. Upon binding of the active GR complex on the 5′UTR of the mRNA, certain proteins are recruited and the HRSP12 (heat-responsive protein 12) endoribonuclease plays an essential role in the GR-mediated mRNA degradation [46] (Fig. 3).

## Factors influencing glucocorticoid action in breast cancer

The mechanisms behind different GC responses have been first investigated in conditions where GCs are frequently used, such as chronic inflammatory diseases and hematological malignancies [47, 48]. Different escape mechanisms have been reported and extensively reviewed by, e.g., Scheijen et al. 2019, Ramamoorthy & Cidlowski 2013, and Ciato et al. 2020 [35, 49, 50]. Due to the strong cell type and context dependency of GCs, regarding breast cancer, the potential role of GC usage has been recently challenged by experimental data [7]. Therefore, in the following paragraphs, the mechanisms of altered GC response and context-dependency are summarized (Fig. 2).

### GR encoding gene (NR3C1) level: sequence and copy number variations

Several studies have linked molecular changes (including expression alteration, copy number variations, and single-nucleotide sequence polymorphisms) of the GR encoding (*NR3C1*) gene to the development of various solid cancer types including breast cancer [51-56].

#### Loss of heterozygosity (LOH) and sequence variations (SNPs) of the *NR3C1*

The tumor-specific loss (CNV, copy number variation) of the GR gene locus was implicated in colon carcinogenesis [53] and in pituitary corticotropinomas, where it was linked to the decreased GC response [52]. However, studies investigating germline and somatic CNVs failed to report a loss of *NR3C1* coding regions associated with breast cancer risk and prognosis [57, 58] (Fig. 2a). However, D5S207, a highly polymorphic dinucleotide repeat located near the *NR3C1* locus, was associated with sporadic breast-cancer development in a Caucasian population [56].

Several pathogenic GR sequence variants were characterized, leading to impaired GR transactivation through negative-dominant effects upon the wild-type receptor, which resulted in a reduced GC response [59]. However, it has been suggested that *NR3C1* mutations were infrequent in GC resistant patients [35].

Apart from pathogenic variants, relatively frequent GR polymorphisms have been shown to influence the GC response. Indeed, two sequence variations (the missense variant N363S and the intronic *Bcl*I) are related to increased sensitivity to GCs; therefore, germline carriers of these variants developed more frequent side effects upon GC treatment [55, 60]. ER22/23EK is associated with decreased transcriptional activity of the GR [35]. Another sequence variant, A3669G, is located within the 3’ UTR of GRβ and results in increased mRNA stability and enhanced GRβ expression [61]. Interestingly, genome wide association studies (GWAS) related to either breast cancer or clinical treatment of breast cancer have not identified any significant associations with GR sequence variants [62, 63].

Therefore, GR sequence variants themselves probably do not represent clinically relevant factors in breast cancer development or conventional treatment. However, due to its context-dependent function, their relation to GC treatment response needs to be further evaluated.

### GR expression on mRNA level—the role of GRβ and other isoforms

#### GR expression in breast cancer has been associated with disease progression

GCs are involved in the development of the mammary gland at puberty and during pregnancy [6, 9, 64]. Also, GR expression was detectable and decreasing during carcinogenesis, and the operational glucocorticoid system was reported to influence breast cancer development (Fig. 2b) [9, 64]. Indeed, GR is expressed in more than 60% of all breast cancers and approximately 70% of ER-positive breast cancers [65]. Chronic GC effects lead to the ligand-mediated downregulation of the GRα (autoregulation) which, in turn, leads to a decrease in the GsC response [51].

Elkashif et al. did not report significant differences in GR expression between normal and cancerous tissue, including epithelial and stromal cells [66]. Also, no significant difference in GR expression was observed among molecular subtypes [66]. However, there are significant associations between prognosis and GR expression (Table 1).

**Table 1:** Prognostic relevance of GR in breast cancer

Study	GR detection method (IHC (antibody)/microarray (probe))	Breast cancer type	Number of investigated cases	Main findings	HR	*P* value
Pan et al. 2011	Microarray (probe na)	ER + untreated	ER + *n* = 1024; numbers of GR high and GR low cases were not reported	In ER + patients: high levels of GR expression in tumors were significantly associated with better outcome relative to low levels of GR expression	RFS HR: 0.6	3.00E-02
		ER + + tamoxifen			RFS HR: 0.25	7.70E-08
		ER − untreated	ER − *n* = 354; numbers of GR high and GR low cases were not reported	In ER − patients: high levels of GR expression significantly correlated with shorter relapse-free survival who were treated or untreated with adjuvant chemotherapy	RFS HR: 2.23	1.00E-03
		ER − chemotherapy			RFS HR: 6.83	5.80E-07
West et al. 2016	Microarray (102865_x_at probe)	ER +	GR high *n* = 311; GR low *n* = 191	In ER + patients: high GR expression is associated with improved RFS in early-stage breast cancer patients, independently of tumor PR expression	RFS HR: 0.35	7.80E-14
		ER + /PR-high	GR high *n* = 215; GR low *n* = 104		RFS HR: 0.35	2.30E-07
		ER + /PR-low	GR high *n* = 96; GR low *n* = 87		RFS HR: 0.4	4.10E-06
Shi et al. 2019	IHC (D8H2, rabbit, Cell Signaling Technology)	All cases	GR high *n* = 68; GR low *n* = 71	In breast cancer patients: GR is negatively correlated with the survival rates	NR	1.00E-04
		ER +	GR high *n* = 42; GR low *n* = 43	In ER + patients similar results were found as in TNBC and invasive subtypes	NR	1.00E-04
		TNBC	GR high *n* = 10; GR low *n* = 17		NR	1.00E-04
Abduljabbar et al. 2015	IHC (SC-1003, rabbit, Santa Cruz Biotechnology)	ER −	GR high *n* = 97; GR low *n* = 138	In ER − patients: univariate analysis showed that positive nuclear GR was associated with shorter breast cancer-specific survival	BCSS/DSS HR: 4.09	4.30E-02
		TNBC	GR high *n* = 59; GR low *n* = 89	In TNBC patients: univariate analysis showed that positive nuclear GR was associated with shorter breast cancer–specific survival	BCSS/DSS HR: 4.22	4.00E-02
		*However, Cox multivariate regression demonstrated that GR is not an independent predictor of survival. No association has been found between GR expression and breast cancer–specific survival (BCSS) or distant metastasis-free interval (DMFI) in the whole series or in the ER-positive group*				
West et al. 2018	Microarray (probe na)	TNBC-basal-like 1	GR high *n* = 43; GR low *n* = 128	In TNBC patients: high expression was associated with worse outcome	RFS HR: 1.87	1.30E-02
		TNBC-basal-like 2	GR high *n* = 19; GR low *n* = 56		NR	6.40E-01
		TNBC-mesenchymal	GR high *n* = 44; GR low *n* = 131		RFS HR: 1.65	4.00E-02
		TNBC-luminal AR	GR high *n* = 57; GR low *n* = 145		RFS HR: 1.68	1.50E-02
Elkashif et al. 2020	Microarray (probe na)	ER − , untreated	GR high *n* = 32; GR low *n* = 32	In ER − patients: high GR expression was associated with significantly poorer RFS and OS	OS HR: 2.615	1.96E-02
					RFS HR: 2.553	8.7E-03
	IHC (D8H2, NR, Cell Signaling Technology)	ER − , taxane-free, anthracycline treated	ER − *n* = 105; numbers of GR high and GR low cases were not reported	In ER − patients: high GR expression was associated with improved outcomes in the context of anthracycline-based chemotherapy	RFS HR: 0.446	4.42E-02
		ER − , taxane treated	GR high *n* = 5; GR low *n* = 13	In ER − patients: high GR expression was associated with poor outcome in response to taxane-based chemotherapy	RFS HR: 4.939	2.37E-01
					OS HR: 1.424	7.72E-01
Chen et al. 2015	Microarray (probe na)	TNBC, untreated	GR high *n* = 70; GR low *n* = 70	In TNBC patients: high expression of GR was associated with shorter overall survival	OS NR	4.00E-02
	Microarray (probe na)	TNBC, chemotherapy	GR high *n* = 29; GR low *n* = 30	In TNBC patients: high expression of GR was correlated with shorter metastasis-free survival in patients undergoing chemotherapy	DMFS NR	9.00E-03

RFS: relapse-free survival; OS: overall survival; BCSS: breast cancer–specific survival; DSS: disease-specific survival; DMFI: distant metastasis-free interval; HR: hazard ratio; NR: not reported; ER: estrogen receptor; PR: progesterone receptor; TNBC: triple-negative breast cancer; NA: not available

In ER-positive breast cancer patients, high levels of GR expression in tumors were significantly associated with better outcome relative to low levels of GR expression independently of treatment, and only one study by Shi et al. reported different results (Table 1) [ 67-69].

In ER-negative breast cancer, however, the opposite effect of GCs was observed, and GR expression was associated with worse survival (Table 1) and with poor outcome in response to taxane-based chemotherapy. Also, this study showed that high GR expression was associated with improved outcomes in the context of anthracycline-based chemotherapy and therefore suggested the potential predictive role of GR in treatment choice [66].

A recent meta-analysis investigating the prognostic significance of GR expression in different cancers found high heterogeneity among tumor types and suggested no association between GR expression and outcome [70]. Subtype analysis showed that among different cancer types, high GR expression associated with reduced progression-free survival (PFS) in early ER-negative untreated cases, but this correlation was not found in late-stage, chemotherapy-treated cancers [70].

##### Causes behind altered GR expression

The reduced expression of the GRα has been associated with treatment-resistant forms and/or diminished GC sensitivity in breast cancer [71]. Reduced expression of the glucocorticoid receptor due to promoter hypermethylation was observed in breast cancer samples, and particularly, but not exclusively, in ER-positive tumors, but this is likely enough to influence GC responsiveness in the tumor cells [72]. Snider et al. 2019 reported that GR methylation was relatively common in ER-positive tumors [73]. Interestingly, both of the investigated GR promoter regions had prognostic value, but with opposite effects on the outcome [73]. The region-specific GR promoter methylation was an independent prognostic marker for patient survival and identified a subset of ER-positive breast cancer patients with poor prognosis, particularly without tamoxifen treatment [73]. However, the lack of methylation in the promoter of the GR gene in non-metaplastic breast carcinomas indicated that methylation was less likely the reason behind the reduction of GR expression in this type of breast cancer [74]. Oppositely, high GR concentration and GR overexpression affect the receptor’s conformation and location and lead to ligand-free dimerization, thus bypassing dimerization-dependent GR activation [18, 30]. In addition, a high concentration of GCs can induce non-genomic effects through GR-independent mechanisms of action by increasing the level of second messengers, such as inositol-3-phosphate, cyclic adenosine monophosphate, and calcium ions [38, 39].

#### Alternative splicing, different GR isoforms, and the role of GRβ

The GR is encoded by a single gene, *NR3C1*. While exons 2–9 consist of the protein coding region, exon 1 contains three transcription-initiation sites, each of which produce an alternative first exon (1A, 1B, and 1C) [13]. Following transcription, different splice isoforms are generated by alternative splicing. GRα is considered to be the main and most widely expressed isoforms in almost all tissues [13]. Together with GRα, the classical receptor, GRβ is considered as the other main GR isoform. GRα and GRβ are identical up to amino acid 727. GRα consists of 777 amino acids, whereas the 50 carboxy terminal amino acids have been replaced by 15 non-homologous amino acids in the GRβ protein (742 amino acids) [13]. Besides the two main transcripts, additional splice variants have been described which are produced by an insertion of an additional arginine coding codon between exons 3 and 4 (GRγ) by skipping of exons 5–7 (GR-A) or by deletion of exons 8 and 9 (GR-P) [13]. GRγ shows a decreased transcriptional activation (approximately 50% of GRα) (Fig. 2c); however, it is widely expressed among different tissues at a relatively low level representing 4–8% of the total GR [13, 35]. The possible role of GRγ has been suggested to be related to a decreased GC response [6, 35].

In addition to transcription and splicing, the protein translation from mRNA into GR protein also introduces further GR protein isoforms. At least eight GR protein isoforms, termed GRα-A to D (A, B, C1, C2, C3, D1, D2, and D3), are synthetized [75]. Each N-terminal isoform of the GRα represents a functional receptor differing in the transcriptional activity and having distinct transactivation and transrepression patterns (Fig. 2d). In addition, they exhibit different cellular localization, as for instance, the GRα-D isoform resides primarily in the nucleus irrespective of the presence or absence of ligand, whereas other isoforms translocate to the nucleus upon ligand binding [13, 75]. The distinct roles of different N-terminal isoforms are well illustrated by the results of a study where high-throughput gene expression profiling revealed that among the more than 2000 GR target genes, only 189 genes were commonly regulated by all GRα isoforms [75].

Recent results concerning the role of GRβ are summarized in the following paragraphs.

##### Beside N-terminal isoforms, C-terminal isoform (GRβ) of the GR exists

Alternative ***splicing*** at the 3’ end of the primary transcript generates GRα and GRβ isoforms as described above. This is due to the short end of the GRβ that prevents it from binding to GCs. The alternative splicing of GR mRNA is modulated by serine/arginine-rich proteins (SRps) in an antagonistic way [20]. Several factors through modulation of the level of SRps have been demonstrated to regulate GRβ expression [20, 76]. GRβ is widely detectable across tissue types, but usually at a lower concentration [13]. While traditionally it was thought that GRβ mainly localized in the nucleus, was unable to bind any ligand, and modulated GC actions by a dominant-negative effect on the GRα, recent data have suggested a more complex GRβ activity, elegantly summarized by Ramos-Ramirez et al. 2021 [20].

In addition, the cellular localization and subcellular trafficking of GRβ upon dexamethasone treatment seem to be cell-type dependent [20], which is also underlined by regulation of GRβ expression through ***miRNAs*** working in a network [20]. Indeed, functional studies have demonstrated that some miRNAs have a direct effect through decreasing or increasing GRβ expression by targeting directly GRβ 3’UTR, while others regulate GRβ expression indirectly by the downregulation of GRα [20].

In contrast to GRα that mediates the classic GC actions, the GRβ isoform is proposed to be responsible for the impairment of GRα activities (see further details below). In line with this, studies have indicated that reduction of the GRα:GRβ ratio contributes to decreased glucocorticoid action [35], emphasizing the potential role of GRβ in the context-dependent GC action. Several ***mechanisms of GRβ action*** have been proposed. It acts as a negative regulator of the GRα isoform (i.e., dominant-negative effect on GRα) by binding to GRE in a ligand-independent way. Upon GRE binding, it does not induce transcription, hence it competes with GRα or it forms an inactive heterodimer with GRα [9, 18, 20, 35]. GRβ also has been shown to bind RU486 (mifepristone), a synthetic GC antagonist, leading to transcriptional changes independently from GRα. Based on this, it can be hypothesized that GRβ may be able to bind other ligands (unknown molecules or endogenous steroids) as well. In addition, the intrinsic activity of the GRβ isoform (in the absence of the ligand) has been also clearly demonstrated by in vitro and in vivo experiments where GRβ exerted transcriptional activity on several genes, including some with GRE-containing promoters and on non-GC-regulated genes [9, 18, 20, 35].

Related to breast cancer, there is a lack of information regarding the implication of GR splice variants and N-terminal protein isoforms [6, 35]. The clarification of the role of GRβ in breast cancer development and progression would be high priority, but due to the sequence similarity between GRα and GRβ, it is difficult to discriminate the two transcripts (the GRβ specific sequences were not included in earlier microarray studies) and proteins. For this, a highly specific GRβ antibody development would be necessary.

### Posttranslational modifications of GR protein

Different posttranslational modifications also play a significant role in regulating GR activity (Fig. 2e). Several extensive reviews focused on the detailed analysis of these modifications (phosphorylation, ubiquitination, SUMOylation, acetylation, methylation, nitrosylation, nitration) [13, 18, 35, 77]. These all have been described to modulate GR transcriptional activity, stability, and localization; however, in the literature, only GR phosphorylation has been investigated related to breast cancer.

GR phosphorylation seems to be enough for receptor activation [78, 79]. While the phosphorylation of some serines is dependent on ligand binding to GR, others can also be phosphorylated in a ligand-independent manner [13]. Some are phosphorylated by mitogen protein kinases (MAPK), cyclin-dependent kinase (CDK), glycogen synthase kinase-3 (GSK-3), and c-Jun N-terminal kinase (JNK), representing crosstalk with other signaling pathways [13, 80, 81]. Also, the phosphorylation of individual serine residues may potentially have effects on the subcellular localization [12, 13], as it decreases the half-life of the GR by promoting a rapid turnover [13, 82, 83] and hyperphosphorylation of GR might account for the glucocorticoid resistance that is observed during the GS/M phase of the cell cycle [13, 84]. In breast cancer, the GR phosphorylation at Ser134 was mandatory for the interaction with PELP1, and it was suggested that targeting phospho-Ser134 GR in certain cases of triple-negative breast cancer may be a useful therapeutical strategy [85, 86].

### GR activation—the role of ligand availability and ligand type

In the presence of an increased amount of GR ligand, the GR monomers are removed from their half sites and instead dimer formation and assembly on classical GREs occurs. Under normal corticosterone levels in mice, GR is preferentially bound to DNA as a monomer rather than as a dimer [25]. Prolonged exposure to GCs increases the expression of FKBP5, which impairs cytosolic GR binding capability and therefore the ultra-short negative feedback loop on GR sensitivity [87]. In breast cancer, unliganded GR was described to display a protective role, as it bound to the promoter region of the *BRCA1* gene, upregulating its expression in non-malignant mammary cells [32]. The presence of GC induced a loss of GR recruitment to the *BRCA1* promoter with a concomitant decrease in *BRCA1* expression [31, 32].

Indeed, increased circulating GC level upon chronic stress has been associated with cancer progression, including breast cancer [64, 88]. An in vivo animal model suggested that rats exposed to chronic stress accompanied by increased GC levels developed more aggressive mammary tumors [89]. Also, cancer-promoting systemic inflammation is a fundamental characteristic of malignant tumors with relevance to the tumor microenvironment too [90].

Type of ligand influences the genomic response upon GR binding through regulating nuclear translocation speed (Fig. 2f) [18]. Classical GR ligands are suggested to induce NLS-1 exposure, leading to rapid (within 4–6 min) nuclear translocation, and unliganded GR shuttling occurs also via NLS-1. However, NLS-2, which is strictly ligand dependent, results in a ten times slower nuclear transport [45–60 min] [18, 91-93]. In contrast to classic GR ligands, a specific breast cancer–associated cholesterol metabolite (6-oxo-cholestan-3β,5α-diol or OCDO) may shift the role of GR toward oncogenesis [94]. In breast cancer samples, higher levels of OCDO and its synthesizing enzymes compared to normal tissues were detected and correlated with worse prognosis [94]. This oncometabolite is able to bind to GR and regulate a different set of genes or lead to opposite expressional changes of the same genes [94]. In addition, it promoted breast cancer cell proliferation in vitro and in vivo independently of ER by activating the nuclear localization of GR [94].

### GR regulatory feedback controls

There are GR target genes which, besides being regulated by GR itself, also regulate GR via negative or positive feedback processes (Fig. 2g), for instance, the loss of B-cell translocation 1 gene (*BTG1*), a critical determinant of GC-induced apoptosis, both by decreasing GR expression and by controlling GR-mediated transcription [95]. *BTG1* was reported to be weakly expressed in primary breast tumors and lymph node metastases compared to benign breast tumors and normal human breast tissues [96], although subtype analyses were not presented. In breast cancer cells (MCF-7 and MDA-MB-231), overexpression of *BTG1* inhibited cell proliferation, induced G0/G1 cell cycle arrest, and promoted apoptosis. Kamalakaran et al. identified differential methylation of CpG islands proximal to *BTG1* in luminal breast cancers differing from non-luminal subtypes that could identify relapse risk independent of other clinical variables [97].

Serum and glucocorticoid-inducible kinase 1 (*SGK1*) is another GC target gene regulated by a wide spectrum of stimuli [98]. It is involved in the regulation of multiple physiological and pathophysiological processes including tumor development, as summarized by Zhu et al. 2020 [98]. Besides inducing expression, high GC levels increase SGK1 activity by phosphorylation, which in turn causes the phosphorylation, hence nuclear translocation of the GR [87]. This implies that *SGK1* is not only a downstream target of GR signaling, but also exerts positive feedback on GR activation [99]. Since then, several lines of evidence proved the role of SGK1 in breast cancer, as *SGK-1* overexpression was frequently detected [100, 101], and activation or upregulation of *SGK1* was implicated in proliferation and metastatic ability of breast cancer [102-104].

### Crosstalks among nuclear receptors and GR in breast

The interaction of GR with the other steroid receptors has been described and it is involved in the control of breast tissue homeostasis (Fig. 2h). Extensive crosstalk between GR signaling and other steroid receptors, including ER, PR, androgen receptor (AR), and mineralocorticoid receptor (MR), have been described in breast cancer in detail [39, 64, 105-107].

#### GR–ER crosstalk

Most (70–75%) breast carcinomas express ER [3]. Its presence is important as it is not only an independent prognostic, but also a predictive marker of response to therapy [3].

ER crosstalk with both PR and AR are the best-characterized models of nuclear receptor interactions in breast cancer, but lately, the interaction of ER with GR has been emerged [105-107].

It has been shown that GR and ER coactivation enhanced GR binding to both GRE and estrogen responsive element (ERE), leading to an increased expression of pro-differentiating genes and negative regulators of pro-oncogenic Wnt signaling, and a decreased expression of epithelial–mesenchymal transition (EMT)-related genes [68]. As GR and ER were shown to co-occupy the same genomic nuclear receptor responsive regions, GCs antagonized estrogen-stimulated endogenous ER target gene expression and estrogen-mediated cell proliferation [68, 108, 109]. This regulation is due to several mechanisms. First, GR could displace ER and its coactivator at the ERE either by direct recognition of ERE or through indirect binding to ERE with other factors such as AP-1, thus antagonizing ER activity [108, 110]. Second, ligand bound GR suppresses the association between ER and chromatin at the enhancer region of E2-induced pro-proliferative genes, subsequently reducing their expression [111]. In this process, GR sumoylation at certain positions is required for GR recruitment to the ER enhancer, and consequently for the repression of the estrogen-related transcriptional program in a target gene selective manner [106, 112]. In addition, another mechanism of GR–ER interaction has been also discovered, termed as assisted loading [110]. Upon induction, GR can modulate access of ER to specific DNA sites by reorganization of the chromatin configuration, thereby assisting the binding of ER [110]. It has been shown that this ER–GR crosstalk could function in both directions: GR reprograms accessibility for ER and ER modulates the chromatin landscape for GR access [105, 110]. Interestingly, it was also revealed that despite the same binding site of the two receptors, they did not compete for the binding site due to their rapid binding kinetics on chromatin [106, 113]. Another mechanism of interplay between GR and ER signaling is that GCs decreased free estrogen levels through GR-mediated activation of estrogen sulfotransferase [114].

Besides the assisted loading, estrogen also influences GC action. It was proved to induce dephosphorylation of GR, decreasing its activity on target genes involved in cell growth arrest [115], and ER antagonists could lead to enhanced proteasomal degradation of GR via E3 ubiquitin-protein ligase Mdm2 (mouse double minute 2 homolog) [116]. Indeed, literature data showed that GR expression was higher in ER-negative breast cancer cell lines compared to ER-positive ones, indicating a reciprocal inhibitory action between GR and ERs [117].

This GR–ER crosstalk manifested as an improved relapse-free survival in ER-positive tumors, and GR was related to a favorable outcome, while low GR expression was associated with high Ki67, p53, and CD71 expression in ER-positive breast cancer [65, 68]. This correlation occurred irrespective of tamoxifen treatment or PR expression level [67, 68].

In the absence of ER, ligand bound GR binds to the GREs of several pro-tumorigenic genes driving drug resistance and progression in TNBC [7, 67, 118]. Therefore, GR activation leads to gene expression pattern related to tumor cell survival, cell migration, and invasion [7, 67, 118]. Transcriptome analysis and in vitro experiments suggest cellular processes, such as EMT, chromatin remodeling, and epithelial cell/inflammatory cell interactions in the involvement of GR in the aggressive behavior of ER-negative breast cancer [7, 67, 118]. Without ER coactivation, GR triggers several oncogenic signaling pathways, such as Wnt and Hippo, *KLF5* (Kruppel-like factor 5) prosurvival transcription factor, and *SGK1* (MKP-1 (MAPK phosphatase-1)) [6, 8]. Even at distant metastatic sites, GR activation due to GC treatment promoted tumor cell colonization and reduced the overall survival by upregulating the expression of *ROR-1* kinase (the receptor tyrosine kinase-like orphan receptor-1) [7].

These biological processes explain findings in hormone receptor negative breast cancer, where higher GR expression was associated with poor prognosis, shorter disease-specific survival, and earlier relapse [65, 67, 118, 119]. Hence, GCs have been shown to represent an increased risk of metastasis in TNBC [8].

Besides direct interaction, indirect crosstalk between GR and ER has also been revealed and reviewed in detail by Paakinaho et al. 2021 [106]. One such indirect crosstalk point is NF-κB signaling. A complex interaction between ER and NF-κB has been described (Franco 2015). A subset of ER enhancers is located in less-accessible regions of the genome that require TNFα signaling to promote NF-κB binding, leading to enhanced chromatin accessibility and subsequent ER binding [120]. TNFα-activated NF-κB and ER together potentiate gene expression associated with proliferation, invasion, and metastasis in breast cancer cells [120, 121]. The constitutive activation of NF-κB was found to be associated with more aggressive ER-positive breast cancers and the development of resistance to endocrine therapy [122]. GR has been also demonstrated to influence NF-κB signaling by suppressing its actions and leading to an inhibition of TNF-α production [123]. In this context, the two receptors act in an antagonistic way that raises a caution of dex usage in ER-positive breast cancer.

In summary, the ER/GR crosstalk has been proposed to be responsible for the differential impact of GR expression and activity across breast cancer subtypes (ER positive vs. TNBC) [105].

#### GR–PR crosstalk

PR are usually co-expressed with ER, probably because PR is an ER target gene [107]. However, in rare cases (< 2% of breast cancer), PR expression occurs without ER positivity [3]. Until now, there is no PR targeted treatment, and PR has a prognostic significance, as it is considered as a biomarker of ER functionality and a predictive marker of response to ER-targeted therapy [105, 107]. Patients with ER + /PR − tumors have worse prognosis compared to ER + /PR + ones, and the overall survival of ER − /PR + cases resemble triple-negative tumors rather than luminal tumors [3]. Indeed, it has been shown for a long time that progesterone-initiated PR signaling contributed to mammary tumorigenesis in murine models [124].

GR expression in breast cancer was positively correlated with the expression of ER and of PR [65]. GR and PR share similar structural characteristics, but while PR specifically binds progesterone, GR was able to recognize both GCs and progesterone with similar affinity [125]. PR is also responsive to GCs and able to bind to GREs and vice versa for GR [105]. Consequently, dex and progesterone agonists both possess GC activity and anti-progestins inhibit GR-mediated transcription [39, 126, 127]. As a result of crosstalk, GC-like effects of progesterone have been shown in some tissues, while progesterone-like effects of GCs in other tissues have also been demonstrated [128].

When both GR and PR were expressed, treatment with either dex or PR agonists resulted in a large overlap of their respective gene regulation [128]. Similarly, both dex and PR agonists downregulated PR expression, suggesting an additional modulation of PR through GR [129]. However, GR was not able to mediate progesterone inhibitory actions. The effect of both dex or PR agonists on half of the evaluated genes was hormone specific, suggesting that GR and PR possess distinct functions, probably through recruitment of differential activators [125, 128]. Negative crosstalk between PR and GR was also demonstrated due to competition for GRE on gene promoters [ 130-132].

In PR + /GR + tumors, GCs mimic the effects of progesterone by inducing growth inhibition, cell spreading, and focal adhesions, effects shown to be mediated by crosstalk with PR [133]. However, in PR − /GR + breast cancer cells, dex induces only a small increase of cell growth and focal adhesions that were not mediated by progesterone [133].

#### GR–AR crosstalk

Androgen receptor (AR) is not routinely assessed in breast cancer patients, despite being expressed in approximately 60–70% of all breast cancer tumors [3, 134]. It is co-expressed with ER in the majority (80–90%) of them, while it is detected only in 15–35% of TNBC cases [3, 134, 135]. Similarly to GR, AR action is also highly context dependent and somewhat controversial. It was dependent on the co-expression of ER, the relative AR/ER expression ratio, the menopausal status, and endogenous androgenic/estrogenic hormone levels [105, 107].

AR is able to bind to ERE [134] and the crosstalk of AR with ER have been also intensively discussed in breast cancer by Truong et al. 2018, Kumar et al. 2021, and Paakinaho et al. 2021 [105–107]. Generally, it has been shown that AR has an antagonistic effect on estrogen [105]. AR + /ER + /PR + breast tumors were smaller, had decreased Ki67, and patients had better survival compared to their AR + /ER − /PR − counterparts [136, 137].

Several studies, including a meta-analysis, showed that the expression of AR in women with breast cancer was associated with better overall and disease-free survival irrespective of ER co-expression [134, 138, 139]. In line with this, an AR agonist, but not an antagonist, was shown to inhibit the proliferation and growth of ER-positive breast cancer cells, patient-derived tissues, and patient-derived xenografts (PDX) [139]. The role of AR agonism has been strengthened by others as well [140]. Based on these findings, it was suggested that ligand-activated AR may function as a non-canonical inhibitor of ER [139]. However, during the historic use of androgen treatment, virilizing side effects have limited its clinical utility, as selective androgen receptor modulators (SARMs) with high specificity of binding to AR have the advantage of dissociating the anabolic from androgenic effects, and consequently lack the virilizing effects [107, 139, 141].

However, other studies described that in postmenopausal ER + breast cancer patients, AR expression was not associated with prognosis, and the authors suggested that AR expression may not be an informative biomarker for the selection of adjuvant endocrine therapy [142].

Interestingly, both AR agonists and antagonists have been shown to inhibit growth in ER + preclinical models by inhibiting ER function at a genomic level [107, 134, 139, 143-146]. Recently, it was also suggested that ER and AR are rather cooperative than antagonistic partners of each other [105]. Therefore, antiandrogens have also been investigated in clinical trials either alone or in combination with other chemotherapeutics or targeted therapies [107].

In contrast to ER-positive breast cancer, it was found that high AR expression associated with a poor prognosis in TNBC [138]. Despite initial studies that suggested a potential negative prognostic role of AR, recently, much evidence indicated that AR expression associated with a favorable prognosis in this type of breast cancer [147].

The interaction of GR with AR is poorly investigated in breast cancer; however, it has been described in adipose tissue, liver, and in prostate cancer [105, 106, 148]. In adipocytes, AR agonism potentiated the transcriptional response to GR in in vitro and in vivo experimental models, while GR antagonism had the opposite effect [148]. However, GR transcriptional output of androgen signaling was tissue specific, as in adipose tissue it was partially attributed to decreased 11B-hydroxysteroid dehydrogenase type 1-mediated glucocorticoid regeneration, while in liver, attenuated GR activity was independent of GC levels [148]. Adipose tissue, as the microenvironment of breast cancer cells, has an important role in tumorigenesis. Hence, the AR–GR interaction might be also important in tumor development. In prostate cancer, AR–GR crosstalk also occurs extensively. While AR and GR transcriptional output presents a considerable overlap, GR activation leads to an attenuation of AR-dependent transcriptional programs, hence a partial antiandrogen effect, suggesting the tumor suppressor role of GR in prostate cancer [105, 149]. Therefore, it has been suggested that GR inhibition may be useful together with AR antagonists for treating prostate cancer [105].

#### GC effect on mineralocorticoid receptor (MR)

The genes encoding the GR (*NR3C1*) and the mineralocorticoid receptor (MR encoded by *NR3C2*) are structurally and functionally similar members of the nuclear receptor (NR) subclass NR3C. While GR is expressed ubiquitously, the MR expression pattern is more delimited. However, in breast cancer, MR is also widely expressed in the majority of cases (up to 90%) [150]. MR can bind the mineralocorticoid aldosterone and GCs with similar high affinity [151]. Therefore, in adult tissues the 11β-hydroxysteroid dehydrogenase type 2 (11β-HSD2) catalyzing the interconversion of hormonally active cortisol and inactive cortisone is found predominantly in mineralocorticoid target tissues, kidney, colon, and salivary gland, where it serves to protect the MR from glucocorticoid excess [152]. However, as breast is not a mineralocorticoid target tissue, GCs can have significant effects on MR. The MR-mediated GC effect has been scarcely investigated in breast cancer. Interestingly, high cytoplasmic expression of MR has been associated with a poor survival of ER + /PR + /HER2 − breast cancer patients [153]. In addition, aldosterone mimics the effects of progesterone by inducing significant growth inhibition, cell spreading, and focal adhesions in PR-positive breast cancer cells, and it induces progesterone-like effects by increasing the expression of p21 and decreasing MAPK phosphorylation [133].

### Chromatin remodeling and GRE site accessibility

Condensed chromatin (heterochromatin) structure inhibits gene transcription by hindering the access of the transcriptional machinery to the DNA sequences. In addition, the interaction between gene-specific transcription factors and their DNA responsive elements is also diminished by heterochromatin structure [35]. Binding site availability depends on chromatin state, which is specific for each tissue and cell type [154]. In line with this, available GREs differ among cell types and are determined by the cellular microenvironment, the transcriptional state, and other factors that are also involved in regulating the accessibility of DNA [155].

Conformational transition between opened chromatin structure (euchromatin) and heterochromatin is required for transcriptional activation and efficient transcription, in which the ATP-dependent SWItch/Sucrose Non-Fermentable (SWI/SNF) protein complex is involved. Chromatin remodeling is an essential component of GR-mediated transcriptional regulation, and the SWI/SNF complex is necessary for glucocorticoid-dependent transcription too (Fig. 2i) [156].

Pottier and colleagues have found significant associations between decreased expression of genes for core subunits of the SWI/SNF complex-*SMARCA4* (SWI/SNF Related, Matrix Associated, Actin Dependent Regulator Of Chromatin, Subfamily A, Member 4), *ARID1A* (AT-Rich Interaction Domain 1A), and *SMARCB1* (SWI/SNF Related, Matrix Associated, Actin Dependent Regulator Of Chromatin, Subfamily B, Member 1) and resistance to [157].

A GC-induced drug-resistant phenotype was developed in a SWI/SNF-dependent fashion in solid tumors, emphasizing the role of GCs in the regulation of chromatin remodeling [158]. The elegant study of Prekovic et al. demonstrated that GR activation in lung cancer led to cell dormancy characterized by a decrease in the overall metabolic activity, significant reduction in proliferation rate, and an increase in the G0/G1 phase of the cell cycle, while GCs did not induce apoptosis. This GC-induced, reversible dormant cellular state was accompanied by a diminished response to a large array of anticancer drugs and was dependent on GR-mediated regulation of *CDKN1C* (Cyclin Dependent Kinase Inhibitor 1C) in a SWI/SNF-dependent fashion through long-range genomic regulation of an upstream distal enhancer [158]. In addition, using transcriptomics and chromatin accessibility data of human tumor samples, it has been shown that this mode of regulation occurred in breast cancer as well [158].

Regarding breast cancer, genetic alteration in genes encoding the SWI/SNF family of proteins have been rarely identified (< 1%), and it corresponded to rhabdoid, composite rhabdoid, sarcomatoid, or anaplastic histologic features [159]. Among SWI/SNF subunits, *SMARCD3* (SWI/SNF Related, Matrix Associated, Actin Dependent Regulator Of Chromatin, Subfamily D, Member 3) depletion led to lower proliferation rate and DNA damage accumulation [160]. In line with this, ER + breast cancer patients with low-*SMARCD3* expressing tumors exhibited reduced survival rates [160].

Chromatin remodeling by the glucocorticoid receptor requires SWI/SNF enzyme subunits SMARCA2 and SMARCA4 complexes [161, 162]. GR and SMARCA2 interdependence was also demonstrated as they selectively modulate each other's occupancy and activity [162]. In breast cancer tissues, *SMARCA2* (or *BRM*) and *SMARCA4* (Brahma-related Gene 1, *BRG1*) ATPases were overexpressed, in most cases independently of the hormone receptor status [163]. Knockdown of *SMARCA2* or *SMARCA4* in a triple-negative breast cancer cell line reduced tumor formation in vivo and cell proliferation in vitro, without any indication of apoptosis, senescence, or alterations in cell migration. Combined knockdown of *SMARCA2* and *SMARCA4* indicated that these enzymes promote cell cycle progression through independent mechanisms [163].

SWI/SNF subunits *SMARCE1* (*BAF57*) and/or *ARID1A* (*BAF250*) were also demonstrated to mediate the interaction between GR and the SWI/SNF complex [156]. While the interrelation between GR and SMARCE1 and ARID1A in breast cancer has not been clearly evaluated yet, both SWI/SNF subunits were associated with breast cancer metastasis and breast cancer patient survival [164, 165].

### Intracellular context-dependent regulatory mechanisms—altered signaling pathways

Defective GR receptor binding due to decreased affinity by cytokines, altered nuclear translocation regulated by phosphorylation, abnormalities in the chaperones and co-chaperones of the GR cytoplasmic complex, or excessive expression of interacting proteins of GR are important determinants of glucocorticoid sensitivity and were identified as potential mechanisms behind a decreased GC response in different diseases [33, 52].

Transcriptional and signaling pathway activity changes (altered expression of key apoptotic genes or activation of survival signaling) represent another context by which GR action is regulated (Fig. 2j). Both cell survival, apoptosis disturbance, and their linkage to GR have been reported and reviewed in detail in breast cancer [40, 50].

For instance, GR and p53 are in reciprocal interaction in breast cancer [39]. P53 was demonstrated to inhibit the binding of GR to GREs (including SGK-GRE) and, on the other hand, p53 stimulates the promoter activity of SGK. In addition, activated GRs have also the potential to suppress the p53 transactivation indicating a mutual interference, through their direct interaction [39, 166].

Other data demonstrated that NF-κB is required for the dex-related protective effect against TNF-α-mediated cell death and correlated with lack of degradation of the anti-apoptotic protein c-IAPI in breast cancer cells [39, 167, 168].

Similarly, pathogenic variants of ***CREBBP*** (CREB Binding Protein) also led to decreased GC response, as GC-responsive genes are under the transcriptional control of CREBBP targets [169]. In breast cancer, elevated expression and gene amplification were described, especially in luminal A and B types [170]. In addition, patients with high *CREBBP* expression had better disease-free survival than the low gene expression group. Therefore, the significance of *CREBBP* as a new therapeutic target in hormone-positive breast cancer was suggested [170]. In addition, in ER-negative breast cancer cell lines, CREBBP exhibited a proliferative effect [171]. These findings are in line with an opposite association of GR with regards to outcome in hormone positive and negative breast cancer cases, emphasizing the GR–CREBBP interaction.

A recent integrative genomic analysis, in addition to already published mechanisms of glucocorticoid resistance, revealed a further 14 genes (*CELSR2, MAPK13, PARD3, CALN1, DAP, RBMS2, PTTG1IP, FAM13A, TAOK3, DCLRE1A, RASGRF2, FBXO9, GALNT1*, and *TMEM126A*) not previously associated with glucocorticoid resistance in acute lymphoblastic leukemia. Genome-wide orthogonal validation identified CELSR2 (Cadherin EGF LAG Seven-Pass G-Type Receptor 2) as a key mediator of glucocorticoid resistance that was strengthened by ***CELSR2*** knockdown [196]. CELSR2 is a membrane-bound G-protein-coupled receptor and it was shown to be a mediator of non-canonical Wnt signaling. *CELSR2* depletion diminished the GC response by a significant decrease in basal expression of the GR and a robust upregulation of the antiapoptotic gene BCL2, resulting in a lower ratio of proapoptotic BIM/BCL2 protein expression [196]. Hence, the use of the Bcl-2 inhibitor venetoclax restored the sensitivity to GCs in mouse xenograft models [196]. In breast cancer cells, based on immunohistochemical investigation, the role of *CELSR2* in the pathogenesis of human mammary neoplasia was suggested due to the increased cytoplasmic staining compared to benign epithelium cells [197]. Also, differential gene expression patterns of CELSR2 were identified in different breast cancer subtypes, as CELSR2 was downregulated in HER2-positive breast carcinoma compared with HER2-negative cancers [198]. Therefore, the role or CELSR2 can be hypothesized to be related to an altered GC response in breast cancer, but this biological link needs to be further investigated and confirmed.

### Intercellular context-dependent regulation—GC’s effect on the microenvironment

The tumor microenvironment plays a key role in breast cancer tumor growth and response to therapies [172]. GR was detectable in the whole normal breast tissue, including adipocytes and myoepithelial cells surrounding lobular and duct units and the stromal and endothelial cells (Fig. 2k) [6, 74].

Although the role of GCs on the microenvironment in tumor growth has been suggested, the contribution of GCs remains unclear [9, 173]. Due to the abundant expression of GR, GCs have an essential effect on decreased immunosurveillance, the secretion of proinflammatory cytokines, and the inhibition of proliferation of stem cell–like cells [173].

Immune cells present in the tumor microenvironment can be inhibited by GCs, leading to decreased immunosurveillance locally. In breast cancer, GR was found to be overexpressed in the stroma and adipose components of breast cancer, with consequent secretion of proinflammatory lymphokines and growth factors implicated in tumor progression [174, 175]. On the contrary, dex inhibited the proliferation of stem cell–like cells in breast cancer, suggesting a cell-type-specific effect of GCs on the tumor microenvironment [176].

## Clinical implications

The tissue-specific expression of GR itself, its coregulators, and transcription factors result in distinct responses among multiple pathways targeted by a given transcription factor, which may explain the pleiotropic but cell-type-specific action of the GR [6].

The dual role of GCs has been well documented [9]. In animal models, GCs protected against cancer development, and several studies point toward a tumor-suppressive role of GR in epithelial solid cancers [9, 177]. However, GR action in cancer biology appears cancer/cell-type dependent and influenced by treatment [64].

### Molecular links between GC/GR in breast cancer and progression

It has been shown that the GR activity signature (expressional changes) has a stronger association with relapse-free survival (RFS) than GR expression alone [118].

In ER-positive breast cancer, GR activation by GCs has been linked to apoptosis regulation and cell proliferation through inhibition of growth factor signaling, modulation of the expression of apoptotic genes, and by interfering with p53 function [108, 112, 125].

In ER-negative breast cancer, the activation of GR was associated with poor prognosis, supporting cancer growth and metastasis, and aggravating clinical aggressiveness [65, 67, 118, 119]*.* In TNBC, GC-regulated genes associated with drug resistance, and with unfavorable clinical outcomes [119]. GR activation was also linked to epithelial-to-mesenchymal transition (EMT), cell adhesion, and inflammation pathways, and it was associated with relapse despite administration of adjuvant chemotherapy [67, 118]. Furthermore, GR activation was protective against apoptosis both in vitro and in vivo [178, 179]. Recently, Obradovic et al. 2019, using both patient-derived and TNBC cell line–derived xenograft models, presented that GR activation increased breast cancer heterogeneity and metastasis. Increased GC levels during cancer progression augmented colonization and reduced survival of animal models of ER-negative breast cancer. The metastasis-promoting effect of GC was attributed, among others, to the increase of ROR1 kinase expression, while the inhibition of ROR1 reduced metastatic outgrowth and prolonged survival in murine model [7]. In another study on TNBC, GR modulation by using mifepristone (non-selective GR antagonist) suggested that mifepristone pre-treatment could be a useful strategy for increasing tumor cell apoptosis in chemotherapy-resistant GR positive TNBC; therefore, it can have a beneficial effect on tumor progression [180]. Indeed, while mifepristone alone had no effect on tumor cell viability or clonogenicity, the addition of mifepristone to dexamethasone/paclitaxel treatment significantly increased cytotoxicity and caspase-3/PARP cleavage. Mifepristone also antagonized GR-induced *SGK1* and *MKP1/DUSP1* gene expression while it significantly augmented paclitaxel-induced GR-positive MDA-MB-231 xenograft tumor shrinkage in vivo [180]*.*

Interestingly, the dose of administered GC seems to also have an important role in progression. Low-dose dex suppressed tumor growth and distant metastasis in both ER-positive and TNBC xenograft mouse models, while administration of high-dose dex enhanced tumor growth and metastasis [181]. In functional assays, dex inhibited cell adhesion, migration, and invasion in a dose-dependent manner. These effects were partly through the induction of miRNA-708 and subsequent Rap1B-mediated signaling in TNBC, while in ER-positive tumor cells, dex also suppressed cell migration, however, independently from miRNA-708-mediated signaling [181].

### Context-dependent response to therapy

GCs have been demonstrated to induce both chemosensitivity and chemoresistance in breast cancer. In breast cancer cell lines, GCs (dexamethasone, dex) have been described to act as a ***chemosensitizer*** [182, 183]. Indeed, dex pre-treatment in a murine-human cancer xenograft model significantly increased anti-tumor activity of several cytotoxic drugs, leading to a significant decrease of cell proliferation and consequential tumor volume, along with increased apoptosis [182, 184]. This was attributed to GC’s TNFα-inducing and anti-angiogenic effect, leading to apoptosis and enhancement of the chemotoxic drug effect on cancer cells [64, 182, 184]. On the other hand, GCs were demonstrated to induce ***chemoresistance*** as well. Studies demonstrated that GCs specifically inhibited chemotherapeutic drug effects by decreasing apoptosis resulting in larger tumor volumes in a human breast preclinical model [178, 179, 185, 186, 187, 188]. Transcriptional changes indicated the upregulation of anti-apoptotic genes such as BCL-XL, BAK, or SGK-1 [178, 179, 186, 189]. Through an extensive literature review, Vilasco et al. concluded that the nature of drug combinations and time course of GC administration might have a crucial effect in the dual effect of GCs [64]. While sequential administration of dex and chemotherapeutic agents in vivo could result in effective anti-tumoral properties, especially when dex was administered at least 12 h before the cytotoxic drugs, concomitant administration could be deleterious or have a weak effect [178, 179, 182, 184, 188, 190, 191].

Several factors can be hypothesized behind context-dependent GC action and response to therapy in breast cancer. These can be tissue specific (cell type or normal vs. tumor cell) and tumor specific (heterogeneity in space and time). Indeed, there are findings demonstrating GC-induced cell-type specific pro- and anti-apoptotic signaling behind different roles of GC in therapy response [192].

Clinical data support the role of GCs in response to chemotherapy. Lin et al. investigated the impact of GC use on survival in two cohorts of breast cancer patients [193]. They found that in the non-chemotherapy cohort, GC use was associated with more axillary lymph nodes, higher stage, and histological grades of II or III, while high-dose GC administration was associated with shorter overall survival in univariate analysis but not in multivariate analysis [193]. In the anthracycline cohort, multivariate analysis showed that GC use at each dose level was significantly associated with longer breast cancer–specific and overall survival. The associations were significant in both ER-positive and ER-negative subgroups for breast cancer–specific survival, and in ER-negative subgroup for overall survival [193].

Based on the previous findings, probably ER has the most important role in GR context dependency due to nuclear receptor crosstalk. In addition, phenotype switch (losing or gaining, e.g., ER) during tumor progression also could result in change in GC response through which it influences response to chemotherapy. During tumor development or progression mutations, epigenetic and metabolic changes can occur. Therefore, region-specific GR promoter methylation or changes in SWI/SNF subunits could influence GC response which are already reported to have prognostic relevance. Alternative splicing and the ratio of different GR isoforms definitely change the response to GC by modulating the balance between GR activating (GRα) and inhibiting (GRβ) function. Posttranscriptional changes of the receptor, increased circulating GC level (e.g., upon chronic stress or treatment), and the presence of oncometabolite OCDO also influences the context-dependent role of GR in oncogenesis.

The context-dependent GC response was also proved by Cairat et al. who presented that systemic GC use represented that breast cancer risk may differ by tumor subtype and stage [194]. According to their findings, GC exposure was not associated with overall breast cancer risk; however, it was associated with a higher risk of in situ breast cancer and a lower risk of invasive breast cancer [194]. In addition, in invasive breast cancer, GC use was inversely associated with ER positivity and with the risk of stage 1 or 2 tumors but positively associated with the risk of stage 3 or 4 breast cancers.

### Approaches targeting GC’s effect in breast cancer

#### Classical GCs, dexamethasone

GCs were reported to be effective in preventing chemotherapy-induced nausea and vomiting during the treatment of epithelial tumors, including breast cancer. Unlike in hematological malignancies where GCs lead to cell death, in epithelial cancers and breast cancer, GCs seem to inhibit apoptosis that has been proposed to interfere with the effects of chemotherapy [185, 195].

In ER-positive breast cancer, GCs inhibited cell migration of several epithelial cancer types, including the MCF10A non-tumorigenic epithelial cell line and estrogen-receptor positive T47D breast cancer cells [188, 195, 196].

However, the pre-treatment with dex significantly attenuated the therapeutic efficacy of paclitaxel on human tumor xenografts established from transplanting human ERα-negative BCs into nude mice [179, 197–199]. In addition, highly metastatic hormone receptor–negative breast cancer cells showed decreased cell migration and glucocorticoid sensitivity compared to parental cells with an accompanying decrease in GR expression and decreased glucocorticoid-responsive gene expression [200]. Obradovic et al. 2019 demonstrated that in TNBC, GCs (dex) may promote increased breast cancer heterogeneity and metastasis [7]. These observations challenged the unconditional use of dex in breast cancer patients.

However, results of clinical studies could not entirely verify these preclinical findings. A systematic review investigated the clinical effect of glucocorticoids on non-hematologic malignancies, including 54 randomized controlled trials, one meta-analysis, four phase l/ll trials, and four case series [201]. Results showed that GC monotherapy exhibited modest response rates in breast cancer. The addition of GCs to either chemotherapy or other endocrine therapy in advanced breast cancer resulted in an increased response rate, but not increased survival. Therefore, the authors concluded that while GC monotherapy has some benefit, the addition of glucocorticoids to other therapies does not change the long-term outcome in advanced breast cancer [201].

Also, a Danish nationwide prospective cohort study found no evidence of an effect of GC use on breast cancer recurrence [202].

As expected, the association of GC with therapy response was also context-dependent. Elkhasif et al. 2020 reported that while their findings confirmed the previous observations that high GR expression was associated with poor outcome in response to taxane-based chemotherapy, high GR expression was associated with improved outcomes in the context of taxane-free, anthracycline-based chemotherapy in TNBC [66].

While GCs are frequent drugs used in clinical trials [39 studies] as adjuvant, or due to GCs’ antiemetic or other effects, no current trial is investigating the context-dependent anti- or pro-tumorigenic role of GC, according to the NIH ClinicalTrials.gov webpage on 2 January, 2022 (Table 2, Online Resource 1) despite to the aforementioned controversial data.

**Table 2:** Studies related to GC’s effect in breast cancer (see details in Online Resource 1)

***NCT number***	***Title***	***Status***	***Phases***
**Mifepristone and breast cancer:**			
NCT02788981	Abraxane (Paclitaxel)® With or Without Mifepristone for Advanced, Glucocorticoid Receptor-Positive, Triple-Negative Breast Cancer	Recruiting	Phase 2
NCT05062174	Targeting Progesterone Signaling for Breast Cancer Prevention in BRCA1 Carriers: A Pilot Study	Not yet recruiting	
NCT01138553	Preoperative Testing of the Anti-Progesterone Mifepristone in Early Stage Breast Cancer	Terminated	Early phase 1
NCT01493310	Nab-paclitaxel (Abraxane) With or Without Mifepristone in Patients With Advanced Breast Cancer	Completed	Phase 1
NCT01898312	BRCA1/2 and Effect of Mifepristone on the Breast	Recruiting	Phase 2
NCT02014337	Mifepristone and Eribulin in Patients With Metastatic Triple Negative Breast Cancer or Other Specified Solid Tumors	Completed	Phase 1
NCT02046421	Carboplatin, Gemcitabine Hydrochloride, and Mifepristone in Treating Patients With Advanced Breast Cancer or Recurrent or Persistent Ovarian Epithelial, Fallopian Tube, or Primary Peritoneal Cancer	Completed	Phase 1
NCT02651844	Mifepristone for Breast Cancer Patients With Higher Levels of Progesterone Receptor Isoform A Than Isoform B	Completed	Not applicable
NCT03225547	Study of Pembrolizumab and Mifepristone in Patients With Advanced HER2-Negative Breast Cancer	Active, not recruiting	Phase 2
NCT05016349	Investigating the Potential Role of a Novel Quadrate Combination Therapy Mifepristone(Antiprogestrone), Tamoxifen, Retinoic Acid and Cannabidiol ( Selective Cyp 26 Inhibitor) for Treating Early Breast Cancer	Not yet recruiting	Phase 3
**OCDO related studies:**			
NCT02863900	Characterization of the Cholesterol-Epoxide Pathway Deregulation to New Therapeutic Perspectives in Breast Cancers. Occurrence of the Deregulations of CE Metabolism in the Different Molecular Subtypes of BC	Unknown status	Not applicable
**SGRM and breast cancer:**			
NCT02762981	Study to Evaluate CORT125134 in Combination With Nab-Paclitaxel in Patients With Solid Tumors	Completed	Phase 1|Phase 2
**Hsp90 and breast cancer:**			
NCT02627430	Talazoparib and HSP90 Inhibitor AT13387 in Treating Patients With Metastatic Advanced Solid Tumor or Recurrent Ovarian, Fallopian Tube, Primary Peritoneal, or Triple Negative Breast Cancer	Withdrawn	Phase 1
NCT00627627	A Study to Evaluate the Antitumor Activity and Safety of IPI-504 in Patients With Advanced Breast Cancer	Withdrawn	Phase 1|Phase 2
NCT01009437	Ritonavir and Its Effects on Biomarkers in Women Undergoing Surgery for Newly Diagnosed Breast Cancer	Completed	Phase 1
NCT02060253	Ganetespib, Paclitaxel, Trastuzumab and Pertuzumab for Metastatic Human Epidermal Growth Factor Receptor 2 Positive Breast Cancer	Completed	Phase 1
NCT02474173	Onalespib and Paclitaxel in Treating Patients With Advanced Triple Negative Breast Cancer	Active, not recruiting	Phase 1
NCT02637375	A Pilot Preoperative Trial of Ganetespib With Paclitaxel for Triple-Negative Breast Cancer	Withdrawn	Not applicable
NCT01246102	AT13387 in Adults With Refractory Solid Tumors	Completed	
NCT01560416	Fulvestrant With or Without Ganetespib in HR + Breast Cancer	Completed	Phase 2
NCT02898207	Olaparib and Onalespib in Treating Patients With Solid Tumors That Are Metastatic or Cannot Be Removed by Surgery or Recurrent Ovarian, Fallopian Tube, Primary Peritoneal, or Triple-Negative Breast Cancer	Active, not recruiting	Phase 1
NCT01677455	An Open-Label Multicenter Phase 2 Window of Opportunity Study Evaluating Ganetespib in Women With Breast Cancer	Completed	Phase 2
NCT01271920	Combination of AUY922 With Trastuzumab in HER2 + Advanced Breast Cancer Patients Previously Treated With Trastuzumab	Completed	Phase 1|Phase 2
NCT00526045	Phase I–II Study to Determine the Maximum Tolerated Dose (MTD) of AUY922 in Advanced Solid Malignancies, and Efficacy in HER2 + or ER + Locally Advanced or Metastatic Breast Cancer Patients	Completed	Phase 1|Phase 2
NCT00803556	Clinical Trial of the Combination of Intravenous Alvespimycin (KOS-1022), Trastuzumab With or Without Paclitaxel in Patients With Advanced Solid Tumor Malignancies or Her2 Positive Metastatic Breast Cancer Who Have Previously Failed Trastuzumab Therapy	Completed	Phase 1
NCT03383692	Study of DS-8201a for Participants With Advanced Solid Malignant Tumors	Active, not recruiting	Phase 1
NCT03890744	ModraDoc006/r in Patients With Breast Cancer	Completed	Phase 2
NCT00637637	External-Beam Radiation Therapy With or Without Indinavir and Ritonavir in Treating Patients With Brain Metastases	Unknown status	Phase 2

#### Selective glucocorticoid receptor agonists and modulators (SEGRAMs)

Selective GC modulators are designed to have the desirable (anti-inflammatory, immunosuppressive, or antitumor) properties of classical GCs without, or with fewer, side effects. SEGRAMs exert their selectivity by triggering only a subset of the GR mechanisms of action [203, 204].

Selective glucocorticoid receptor agonists (SEGRAs) usually have a classic steroid structure, while selective glucocorticoid receptor modulators (SEGRMs) are typically non-steroidal. SEGRMs are able to modulate the activity of a GR agonist and/or may not classically bind the glucocorticoid receptor ligand-binding pocket [203].

It is generally assumed that SEGRAMs shift GR activity toward transrepression to have a better therapeutic index than classical glucocorticoids, although the transrepression versus transactivation concept proved to be too simplistic [8, 203–205].

In ER + breast cancer models, different SEGRAM compounds (CORT125134, CORT118335, CORT108297) decreased the occupancy of the ER at several enhancers, and the displacement of ER from chromatin by the liganded GR inhibited E2-induced proliferation [111]. Interestingly, SEGRAMs inhibited the action of mutant ER as well, raising its potential effectiveness in endocrine therapy-resistant ER + breast cancer [111].

An advantage of CORT125134 is that it exerts its effects by competing with the binding of cortisol to GR. Unlike mifepristone, it has no affinity for the progesterone receptor and thus does not cause antiprogesterone effects.

Interestingly, there is a phase I/phase II study evaluating CORT125134 (relacorilant) in combination with nab-paclitaxel in patients with solid tumors (NCT02762981), including TNBC cases (Table 2). Unfortunately, results were presented related to pancreatic and gynecologic cancer only [206], and the study has been progressed to phase III in patients with metastatic pancreatic ductal adenocarcinoma (NCT04329949).

Another SEGRM, CpdA, also showed a favorable effect on the expression of GR-mediated pro-tumorigenic genes [119]. Namely, in TNBC cells, CpdA regulated only a small number of genes that were not involved in tumorigenesis and therapy resistance. The authors demonstrated that dex- but not CpdA-liganded GR binds to a single GRE, which drives the expression of pro-tumorigenic genes [119].

#### GC antagonists—RU486

In TNBC cells, GR induces genes related to cell survival and migration, and suppresses those related to cell death [7, 200]. In line with this, GR antagonism could reverse this expression pattern, suggesting that combining GR antagonists with chemotherapy may improve the outcome for ER − /GR + patients [118].

Skor et al. reported that the pre-treatment of TNBCs with the GR antagonist mifepristone in parallel to dex and paclitaxel potentiated the cytotoxic efficacy of the chemotherapy by inducing caspase-3/PARP cleavage-mediated cell death, and blocked GR-mediated survival signaling by antagonizing GR-induced SGK1 and MKP1 gene expression [180]. Also, mifepristone suppressed TNBC cancer stem cells by downregulating KLF5 expression [207]. TEAD4 (TEA Domain Transcription Factor 4), a member of Hippo signaling, is a direct target of GR and it was described to be influenced by GR antagonism. TEAD4 acts as an oncogene in breast cancer, and its high expression predicts poor survival [208]. GCs promoted TEAD4 expression levels, nuclear accumulation, and TEAD4 transcriptional activity. While TEAD4 activation by GC promoted breast cancer stem cell maintenance, cell survival, metastasis, and chemoresistance both in vitro and in vivo, it was also completely blocked by cotreatment with mifepristone [208].

Indeed, the GR antagonist RU486 (mifepristone) is currently being registered in 10 clinical trials for breast cancer (NCT02788981, NCT05062174, NCT01138553, NCT01493310, NCT01898312, NCT02014337, NCT02046421, NCT02651844, NCT03225547, NCT05016349), among which 4 are completed, 1 is active, not-recruiting, 2 are not yet recruiting, and 1 has been terminated (Table 2, Online Resource 1).

It was reported that mifepristone pre-treatment decreased MDA-MB-231 xenograft tumor growth [180]. Nanda et al. 2016 also showed that GR is a promising target in TNBCs, as patients with GR-positive and triple-negative tumors responded to the combination of GR antagonism (mifepristone) and paclitaxel (NCT01493310) [209].

Another recently submitted phase III trial (NCT0501634, not yet recruiting) aims to investigate the effect of mifepristone as part of a novel quadrate combination therapy (with tamoxifen, retinoic acid, and cannabidiol as a selective Cyp26 inhibitor) for treating early breast cancer.

As mifepristone is non-selective, it has an antagonistic effect on PR as well, and it was shown to be active in some preclinical hormone-dependent breast cancer tumor models. In a phase II study, the response rate of mifepristone in PR-positive recurrent breast cancer patients who had received no prior therapy was investigated. However, it had minimal activity and only three partial responses were noted for an overall response rate in 10.7% (95% CI: 28%) of the 28 enrolled patients [210].

#### GR co-regulators

As recently suggested, due to its pleiotropic action, targeting GR activity is not a favorable therapeutic option, especially as it is routinely used alongside conventional chemotherapy [6]. However, similarly to ER, where ER co-regulators are thought to contribute to tamoxifen response and resistance [211], targeting co-regulators of GR may potentially serve as a treatment option.

Indeed, as Nourredine et al. suggested, modulating the activity of one (or a subset of) co-regulator(s) could therefore affect GC regulation of only selected GR target genes, and hence selectively promote or inhibit specific GC-regulated pathways [6]. In line with this, it was found that the coactivator activity of a certain GR co-regulator (G9a) was modulated by methylation or phosphorylation. G9a, depending on its posttranslational modification, regulated distinct physiological pathways, including migration of the lung cancer cell line A549 and GC-induced cell death in leukemia [212, 213].

Hsp90, a chaperone protein, is another important GR-interacting partner having the potential to be targeted to influence GR action. Its activity is essential to the folding of the GR into a conformation that allows GC binding and subsequent GR transcriptional activity [198]. The use of an Hsp90 inhibitor resulted in GR degradation and decreased GR-mediated gene expression, and consequently it also sensitized TNBC cells to paclitaxel-induced cell death both in vitro and in vivo [198]. Therefore, the authors concluded that GR-regulated anti-apoptotic and pro-proliferative signaling networks in TNBC were disrupted by Hsp90 inhibitors, thereby sensitizing TNBC to paclitaxel-induced cell death. In addition, they suggested that GR + TNBC patients may be a subgroup of breast cancer patients who are most likely to benefit from adding an Hsp90 inhibitor to taxane therapy [198].

Hsp90 inhibitors are popular drugs, and their preclinical and clinical studies are nicely summarized in asthmatic and rheumatoid diseases [214]. In breast, a systematic review by Zagouri et al. 2013 has also summarized recent advances (19 published studies) regarding Hsp90 inhibitors [215]. Based on initial studies, Zagouri concluded that the greatest clinical activity has been observed on the field of HER2-positive metastatic breast cancer [215]. However, accumulating data suggest that Hsp90 inhibitors may play a significant role in the treatment of triple-negative and aromatase inhibitor-resistant breast cancer [215].

Indeed, among currently registered 16 Hsp90 inhibitor trials in breast cancer [16], 9 are already completed and 3 are active, not recruiting (Table 2, Online Resource 1). A multicenter trial evaluated the Hsp90 inhibitor, ***retaspimycin*** HCL (IPI-504), plus trastuzumab in patients with advanced or metastatic HER2-positive breast cancer, although only modest clinical activity was observed that did not meet criteria for trial expansion [216]. Another compound, ***AUY922***, showed a 22.0% overall response rate and 48.8% of the patients had stable disease among the 41 enrolled patients in combination with trastuzumab in patients with locally advanced or metastatic HER2-positive breast cancer that had been previously treated with chemotherapy and anti-HER2 therapy (NCT01271920) [217]. These data confirmed that HSP90 inhibition in combination with trastuzumab may be a promising strategy in advanced or metastatic HER2-positive breast cancer patients progressing on trastuzumab. Their results were comparable to a ***tanespimycin*** (Hsp90 inhibitor) plus trastuzumab combination, where ORR was 22% and disease stabilization rate was 37% [218]. Similar results were found using ganetespib (NCT02060253) [219] and onalespib (NCT02474173) [220]. ***Ganetespib*** showed stronger anti-tumor activity compared to tanespimycin over a broader range of breast cancer subtypes, including HER2-normal cancer and triple-negative breast cancer (TNBC), with a more favorable safety and toxicity profile [219]. ***Onalespib*** in combination with olaparib has been recently suggested for disease stabilization in patients with *BRCA*-mutated ovarian cancers and acquired PARPi resistance, and in patients with tumors harboring RB-pathway alterations [221].

***Ritonavir*** (NCT03383692), besides being an antiviral agent, inhibits breast cancer growth in part by inhibiting Hsp90 substrates [222] and it is also a dual OATP1B/CYP3A inhibitor. Using ritonavir in combination with trastuzumab deruxtecan in patients with HER2-expressing advanced tumors reduced tumor burden across multiple tumor types, including breast (15/17 cases) [223].

## Conclusion

Although GCs and GR-mediated actions are intensively investigated, their pleiotropic, context-dependent, and cell-type-specific actions are not entirely understood. Their role in breast cancer, despite recent advances, is complex and still unpredictable. To clarify and specify this action of GC/GR system, the level of GR expression, the detection of its splice variants and posttranslational modifications, and its subcellular localization should be further investigated in clinical specimens.

Seemingly, the presence of ER is a key regulator of GR action, and their crosstalk was proposed to be responsible for the differential impact of GR expression and activity across breast cancer subtypes (ER + vs. TNBC). However, controversial preclinical and clinical findings will likely lead to further studies to understand the underlying causes. Also, the high context dependency of GC action represents a principal challenge in the discovery of further connections.

As in breast cancer, GCs are frequently used to mitigate undesirable side effects of conventional chemotherapy, and recent findings that GCs can promote tumor development and metastasis have raised understandable concerns.

Due to this pleiotropic action, as recently suggested, targeting GR activity is not a favorable therapeutic option [6]. This is further supported by the relevance of hierarchical network modeling in cancer drug-target selection [224]. In an interesting study applying hierarchical network structure to biological networks representing genome-scale dynamics, authors were able to faithfully model the therapy response by analyzing FDA-approved drugs compared to drugs that have been rejected [225]. If global transcriptional and protein–protein interactions are considered as a network, genes and proteins in such network represent nodes, and the interactions between them represent edges. Hubs are defined as the top 20% of the highly connected nodes. In a hierarchical network, genes/proteins in the top layer are the master regulators of the network because they influence the whole network through their downstream targets [226]. The core layer is the most abundant layer because it contains most of the HUBs and network motifs (interactions). The core layer plays a central role in the regulation of signal propagation. The bottom genes are located in the third layer, which directly regulates the genes of effector molecules, or they are the effectors of the network [225, 227]. Although nodes in the top and core layers are more likely to be important for the successful survival and adaptation of the organism to its environment, it has been demonstrated that drugs targeting the higher levels of a hierarchical network were more influential [224], while those targeting HUBs are not so effective [224]. In a hierarchical network, GR can be considered as a hub showing typical hub characteristics, such as regulating or co-regulating a high amount of all members of the network [225]. Indeed, GR is regulated by several factors and regulates a significant amount (10–20%) of the whole transcriptome, hence have numerous interactions in a hierarchical network [225]. Therefore, GR targeting therapy in a similar analysis may fall into the not efficient drug category; however, GR targeting in combination could be beneficial.

Therefore, in the use of GC and its agonists in breast cancer, the GR’s context-dependent functions have to be kept in mind. However, GR selective modulators and targeting GR co-regulators probably will be promising and effective therapeutical options, a conclusion that is supported by the current number of clinical trials investigating SEGRAMs or Hsp90 inhibitors.

With the growing body of evidence related to the potential correlation of GR with prognosis and response to therapy, as suggested earlier, the assessment of GR (and other steroid receptors) status in tumor tissue (quadruple and/or quintuple with AR) may add further possibilities for patient’ classification in order to select that best therapeutical approach, i.e., endocrine agonist/antagonist therapies and chemotherapy [3].

## Supplementary Information

Below is the link to the electronic supplementary material.

Online Resource 1 (Supplementary Table 1) GC-related NIH registered trials (downloaded from ClinicalTrials.gov on 2 January, 2022) (XLSX 42 KB)
